# Catheter-associated *Mycobacterium intracellulare* biofilm infection in C3HeB/FeJ mice

**DOI:** 10.1038/s41598-023-44403-0

**Published:** 2023-10-10

**Authors:** Kentaro Yamamoto, Yusuke Tsujimura, Manabu Ato

**Affiliations:** Department of Mycobacteriology, Leprosy Research Center, National Institute of Infectious Diseases, Aoba-cho, Higashimurayama, Tokyo Japan

**Keywords:** Bacteriology, Animal disease models

## Abstract

Non-tuberculosis mycobacterial (NTM) diseases are steadily increasing in prevalence and mortality worldwide. *Mycobacterium avium* and *M. intracellulare*, the two major pathogens of NTM diseases, are resistant to antibiotics, and chlorine, necessitating their capacity to survive in natural environments (e.g. soil and rivers) and disinfected municipal water. They can also form biofilms on artificial surfaces to provide a protective barrier and habitat for bacilli, which can cause refractory systemic disseminated NTM disease. Therefore, preventing biofilm formation by these pathogens is crucial; however, not many in vivo experimental systems and studies on NTM biofilm infection are available. This study develops a mouse model of catheter-associated systemic disseminated disease caused by *M. intracellulare* that reproduces the pathophysiology of catheter-associated infections observed in patients undergoing peritoneal dialysis. In addition, the bioluminescence system enabled noninvasive visualization of the amount and distribution of bacilli in vivo and conveniently examine the efficacy of antimicrobials. Furthermore, the cellulose-based biofilms, which were extensively formed in the tissue surrounding the catheter insertion site, reduced drug therapy effectiveness. Overall, this study provides insights into the cause of the drug resistance of NTM and may guide the development of new therapies for NTM diseases.

## Introduction

Non-tuberculosis mycobacterial (NTM) disease poses a grave threat to global public health as its incidence and mortality rates continue to rise^1–3^. This disease has a higher incidence rate than tuberculosis, which is caused by *Mycobacterium tuberculosis* (*Mtb*), in several developed countries, including USA and Japan^4,5^. NTM disease responds more slowly to treatment, and its severe cases may have a poorer prognosis than multidrug-resistant tuberculosis^6^. Because NTM grows in the natural environment in soil, public baths, shower heads, swimming pools, and urban tap water, we may be constantly exposed to NTM in our daily lives^7–10^. In addition, recent research indicates that the previously ruled out human-to-human transmission of NTM has been proven^11^. Mycobacterium avium complex (MAC) lung disease is the most prevalent pulmonary NTM disease^12,13^, which refers to infections caused by bacilli: *Mycobacterium avium*, *M. intracellulare,* and *M. chimaera*. Although the therapeutic drugs for the infection should be selected based on the drug susceptibility test results, the minimum inhibitory concentration (MIC) of MAC in vitro may not accurately reflect the therapeutic effect, making it difficult to determine the treatment regimens^14–16^.

Chakraborty et al. recently reported that *Mtb* can form biofilms in the lungs of mice and primates, suggesting that its robust biofilms protected it from antimicrobials and the host immune system^17^. Their findings also suggested that biofilms contributed to the latent infection of *Mtb*, thereby exacerbating pulmonary tuberculosis symptoms. Furthermore, NTM bacilli can colonize, and grow on indwelling medical devices implanted into the human body, such as prosthetic joints, peritoneal dialysis catheters, and gastrostomy tubes, even if they are not in the respiratory tract^18–20^. In these cases, eradicating bacilli firmly adhered to artifacts is difficult due to their poor response to medication therapy, and the clinical conditions may worsen, resulting in systemic disseminated NTM disease. Owing to NTM’s slow growth, patients may experience diagnostic delays and inadequate treatment, which can have devastating effects^21,22^. Notably, NTM can survive in any natural environment with water, as it forms biofilms in tap water pipes, supply and drainpipes of swimming pools and public bathhouses, inside shower heads, and in the tanks of household washing machines^23–25^. Matrix-like biofilm formation of *Mtb* and NTM has also been observed under laboratory conditions^26–28^. Recent studies have revealed enhanced biofilm formation in *M. avium* under hypoxic and eutrophic conditions, suggesting hypoxia adaptation in granulomas and links to dormancy^29–31^. Although the layered structures of adherent bacilli are thought to be biofilms comprising the extracellular matrix, the biological properties of NTM biofilm and its scientific evidence in vivo remain unclarified.

Some mouse strains recapitulate key elements of the pathogenesis of human Mycobacterium infection, particularly tuberculosis (TB), at the level of induction of well-organized, hypoxic, and necrotic TB lesions in the lungs^32^. Despite differences in susceptibility to infection and disease manifestations between humans and animals, experimental models such as C3HeB/FeJ mice that present with lung pathology more typical of human TB disease are useful for hypothesis-driven research aimed at understanding TB immunopathogenesis and compound efficacy^32,33^. Interestingly, exposure to an aerosol infection with a rough colony-type strain of *M. avium* leads to a progressive infection characterized by increased bacterial burden in the lung and the formation of necrosis foci in granulomas, similar to those observed in human MAC patients^34^. Therefore, we attempted to develop a new mouse model using C3HeB/FeJ mice to elucidate the role of biofilms in vivo in refractory MAC pathogenesis with necrotic abscesses.

This study focuses on systemic disseminated MAC disease caused by the colonization of indwelling catheters with bacilli. We visualized *M. intracellulare *in vivo using a Lux system as a bioluminescent probe, which is derived from the products of the *luxCDABE* genes in a single operon of *Photorhadus luminescens*. LuxC, LuxD, and LuxE are fatty acid reductases, transferases, and synthases, respectively, which function continuously to oxidize long-chain fatty aldehydes and synthesize fatty acids. Synthesized fatty acids oxidize reduced flavin mononucleotides (FMNH_2_) and emit blue photons with a maximum wavelength of 490 nm^35,36^. Based on the analysis of C3HeB/FeJ mice infected with bioluminescent *M. intracellulare*, we developed a mouse model of systemic disseminated MAC disease with necrotic foci similar to human tuberculous lesions that allowed noninvasive visualization of the changes in the amount and distribution of bacteria due to antimicrobial medication. In addition, the therapeutic effect of anti-NTM drugs was partly reduced due to biofilm-like structure derived from *M. intracellulare* on the indwelling catheters in the peritoneal cavity and peripheral tissues. With the proposed unique mouse model, there is now the possibility of investigating the in vivo role of NTM biofilms.

## Results

### Construction of *M*. *intracellulare* expressing Lux bioluminescence

To noninvasively detect bacilli in vivo, we constructed an integrative plasmid encoding LuxCDABE of *Photorhabdus luminescens*. *M. intracellulare* type strain (ATCC13950) cells were transformed with this plasmid, and the resulting cells expressed the bioluminescence of the Lux system. The MICs of clarithromycin (CLR), ethambutol (EB), and rifampicin (RFP) against *M. intracellulare* with or without the Lux plasmid were determined. Comparing the MICs of the two strains revealed no significant differences, indicating that carrying the Lux plasmid had no significant effect on the antimicrobial susceptibilities of bacilli (Supplementary Table). We also detected a strong linear correlation between the bioluminescent intensity and the number of *M. intracellulare* bacilli harboring the Lux system (Supplementary Fig. S1).

### Imaging of *M*. *intracellulare* in catheter-implanted mice using in vivo bioluminescence imaging system

**Figure 1:**
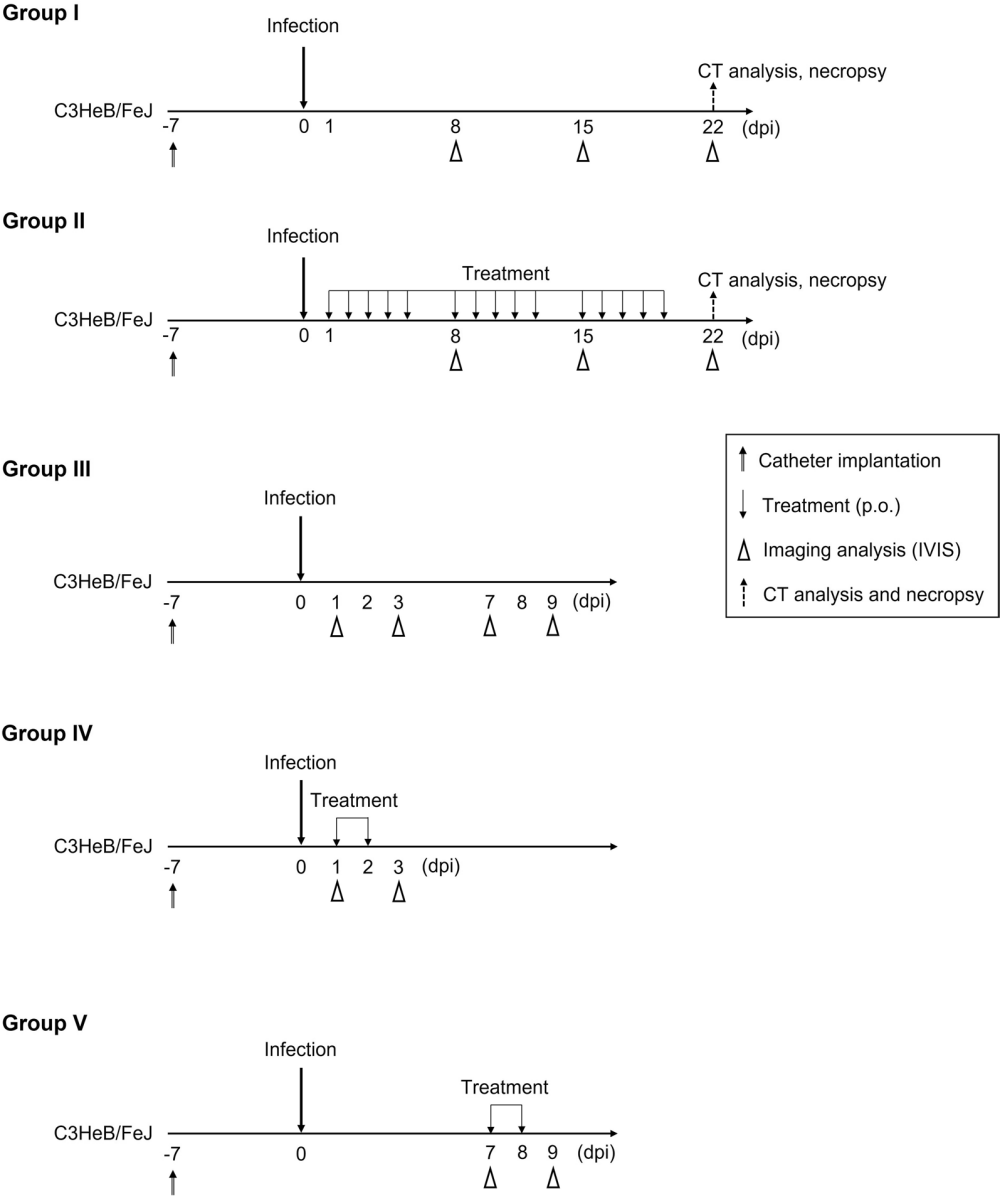
Design of in vivo experiments in this study. Schematic illustration of five in vivo assays (Group I−V), according to animal groups, and time points for analysis. Catheter fragments were implanted into the abdominal region of each C3HeB/FeJ mouse (double-line arrows). These mice were infected by i.p. injection of *M. intracellulare* (10^8^ CFU/mouse). Antimicrobial treatments were performed with clarithromycin, ethambutol, and rifampicin (downward arrows). Bioluminescence in mice was detected using IVIS Lumina (arrow heads). Images were acquired for 300 s and filtered using the smoothing algorithm to remove signal noises. In addition, mice were imaged via CosmoScan FX (dashed arrow).

**Figure 2:**
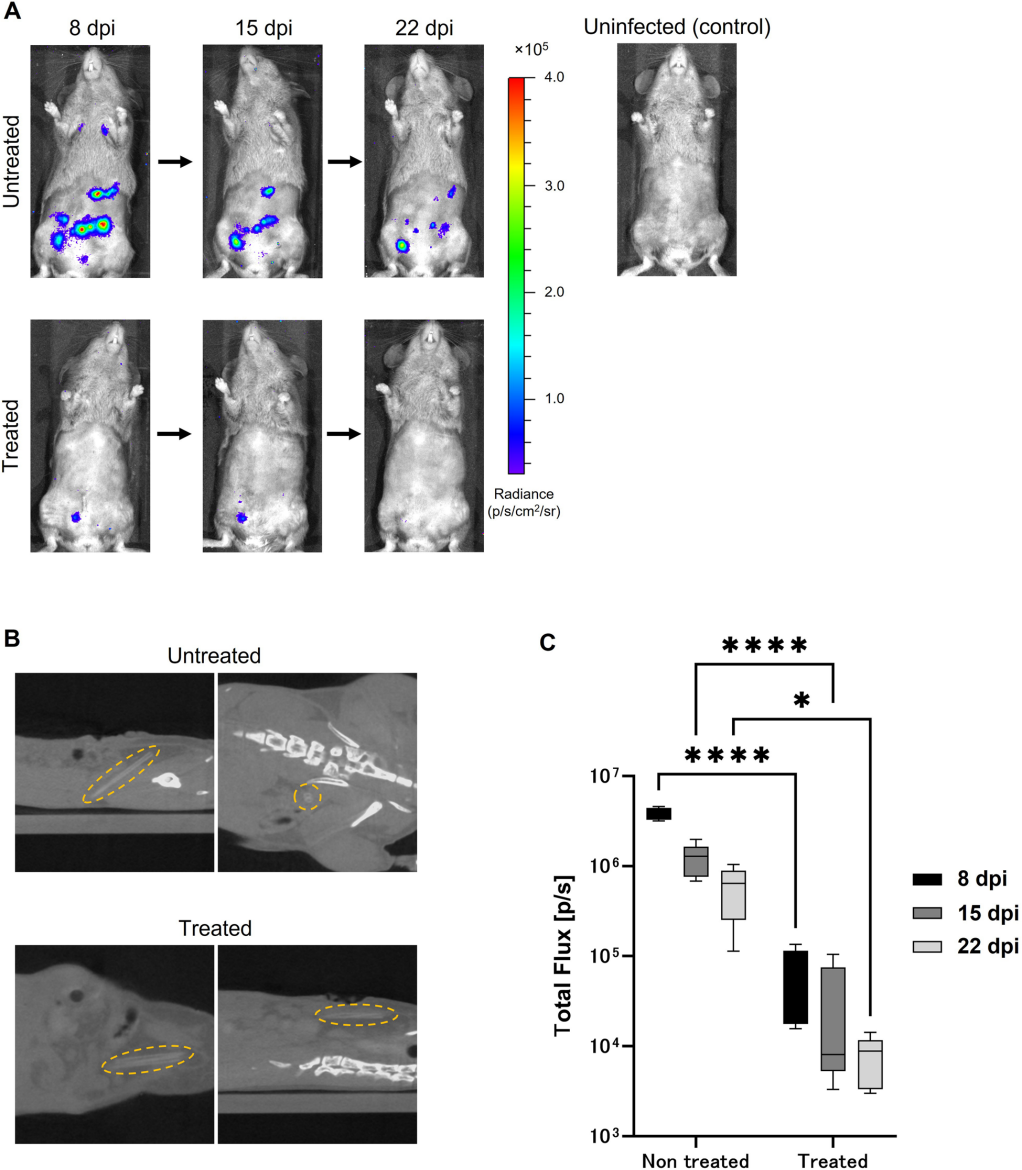
Bioluminescent in vivo imaging of Lux system in mice. (**A**) Representative series of bacterial bioluminescence signals on a color scale superimposed on a grayscale image of a single mouse from untreated (above) and treated (below) with antimicrobials at the indicated time points. The uninfected control mouse is shown at the upper right. The color scale shows photons per second per square centimeter per steradian (p/s/cm^2^/sr). (**B**) The sagittal (left) and coronal (right) CT images from representative *M. intracellulare*-infected mouse at 22 dpi with or without antimicrobial treatment. Dotted circles show the visualized catheter in the peritoneal cavity. (**C**) Calculation and plot of bioluminescent intensity at each time point expressed in photons per second (p/s). Statistical analysis by ANOVA with Sidack’s multiple comparison test: *P < 0.05, ****P < 0.0001.

**Figure 3:**
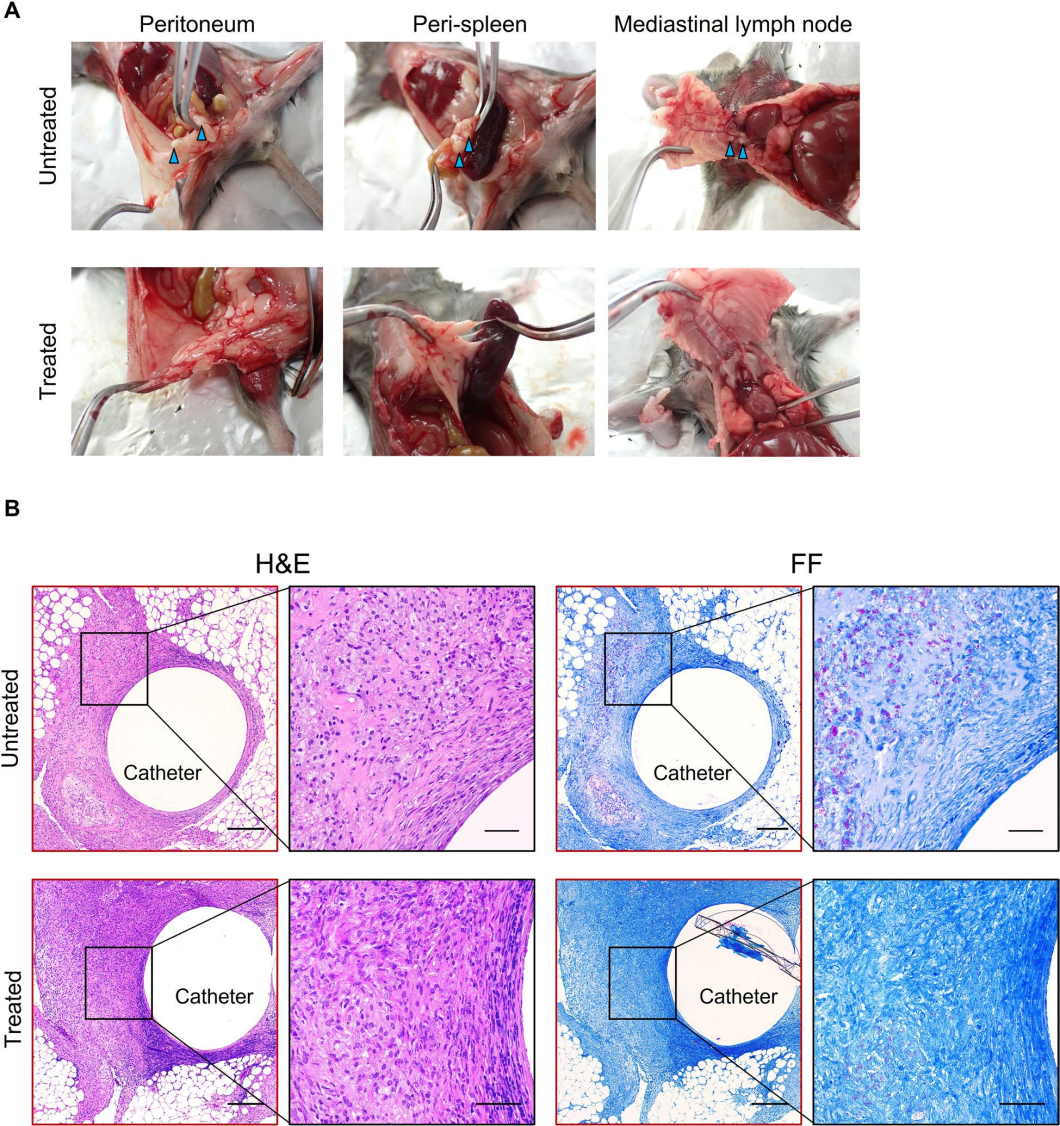
Abscess around a catheter placement in the peritoneal cavity. (**A**) Infected mice were sacrificed 22 days after *M. intracellulare* inoculation and visually inspected for the presence of abscesses (arrow heads). (**B**) Histological study of tissues surrounding the catheter stained by H&E and FF (Fite-Faraco) staining. The left panel (4 ×) shows a wide-angle view of the surrounding tissue with the catheter in the center, and the right panel (20 ×) is a magnified view of the tissue in contact with the catheter. Scale bar = 200 µm (4 ×), 50 µm (20 ×).

To monitor the dynamics of NTM involved in the formation of human peritoneal abscesses, we attempted to construct a mouse model by infecting the abdominal cavity of C3HeB/FeJ mice, but no peritoneal abscess formation was observed (Supplementary Fig. S2). Therefore, catheter fragments were implanted into the right abdominal region of C3HeB/FeJ mice to stabilize bacteria in the abdominal cavity. Seven days after implantation, the mice were infected intraperitoneally with *M. intracellulare* (10^8^ CFU/mouse) expressing LuxCDABE. The infected mice were randomly divided into 5 groups: (I) no treatment and (II) triple-drug treatment with CAM, EB and RFP; each treatment was started at 1-day postinfection (dpi) (“Materials and Methods”, Fig. 1). Treatment for short periods is included in Groups III − V which will be discussed in the last section of the results. While the bioluminescence images of each mouse were acquired at 8, 15, and 22 dpi, the CT images were acquired only at 22 dpi (Fig. 1). In Group I, bioluminescent foci were found until 22 dpi in all intraperitoneal regions of the mice, including the spleen. While the luminescent foci decreased slightly over time, robust luminescence on the right lower abdomen over the indwelling catheter was detected at all time points (Fig. 2A,B). In contrast, no bioluminescence signal was detected in the uninfected control mice (Fig. 2A, upper right). The calculated mean intensities of foci in Group I were 3.8 × 10^6^ ± 6.1 × 10^5^ (mean ± standard deviation (S.D.)), 1.2 × 10^6^ ± 5.1 × 10^5^, and 5.9 × 10^5^ ± 3.5 × 10^5^ photons per second (p/s) at 8, 15, and 22 dpi, respectively (Fig. 2C). In Group I, some large abscesses formed around the peritoneum where the catheter was inserted. Abscesses were also present around the spleen, and the mediastinal lymph nodes were swollen and filled with pus. The location of these abscesses overlapped with the areas of high brightness on in vivo imaging (Fig. 3A). These data indicated that infected *M. intracellulare* diffused and disseminated systemically from the catheter, where bacilli swarmed, and grew as a primary lesion. In Group II, where mice were treated with a three-drug combination, faint bioluminescent foci were observed up to 15 dpi. These foci barely remained on the abdominal region of the mice at 22 dpi. Their mean intensities were 7.1 × 10^4^ ± 5.2 × 10^4^, 3.4 × 10^4^ ± 4.3 × 10^4^, and 7.8 × 10^3^ ± 4.6 × 10^3^ (p/s) at the same time points (Fig. 2C). While abscesses were dispersed around the indwelling catheters in Group I mice, few abscesses were observed in the peritoneum, mediastinum, and other organs in the medication-treated group (Fig. 3A). Based on these data, the effects of medication on bacterial clearance in this mouse model could be clearly compared using bioluminescent imaging, which was statistically significant at each time point (*P* < 0.0001, < 0.0001 and < 0.05, respectively).

### Histopathological analysis of abscesses around indwelling catheters

Here, we performed a histological analysis of abscesses formed in the tissue around the indwelling catheter and peritoneum of *M. intracellulare*-infected mice. Observations revealed granulomatous inflammation surrounding the catheter in concentric circles. This granulomatous inflammation was roughly divided into three layers. Dead inflammatory and fibroblast-like cells were observed in the first layer, where the catheter made contact. Capsule-like structures also formed on the catheter surface. The second layer primarily comprised foamy macrophages, and narrow necrotic nests, and granulomas with neutrophils were also observed. The third layer was a transition from foamy macrophages to an epithelioid cell layer, with neutrophils scattered among the cells (Fig. 3B). Fite-Faraco staining (FF staining), a method for detecting mycobacteria in a specimen, revealed the majority of bacteria in the second layer of foamy macrophage accumulation. However, the sections of the treated group had fewer bacteria around the catheter than Group I. Some necrotic nests were observed, and fibrous tissue was increased in the area in contact with the catheter (Fig. 3B).

### *M*.* intracellulare* formed cellulose-layer on catheters in mouse abdominal cavity

**Figure 4:**
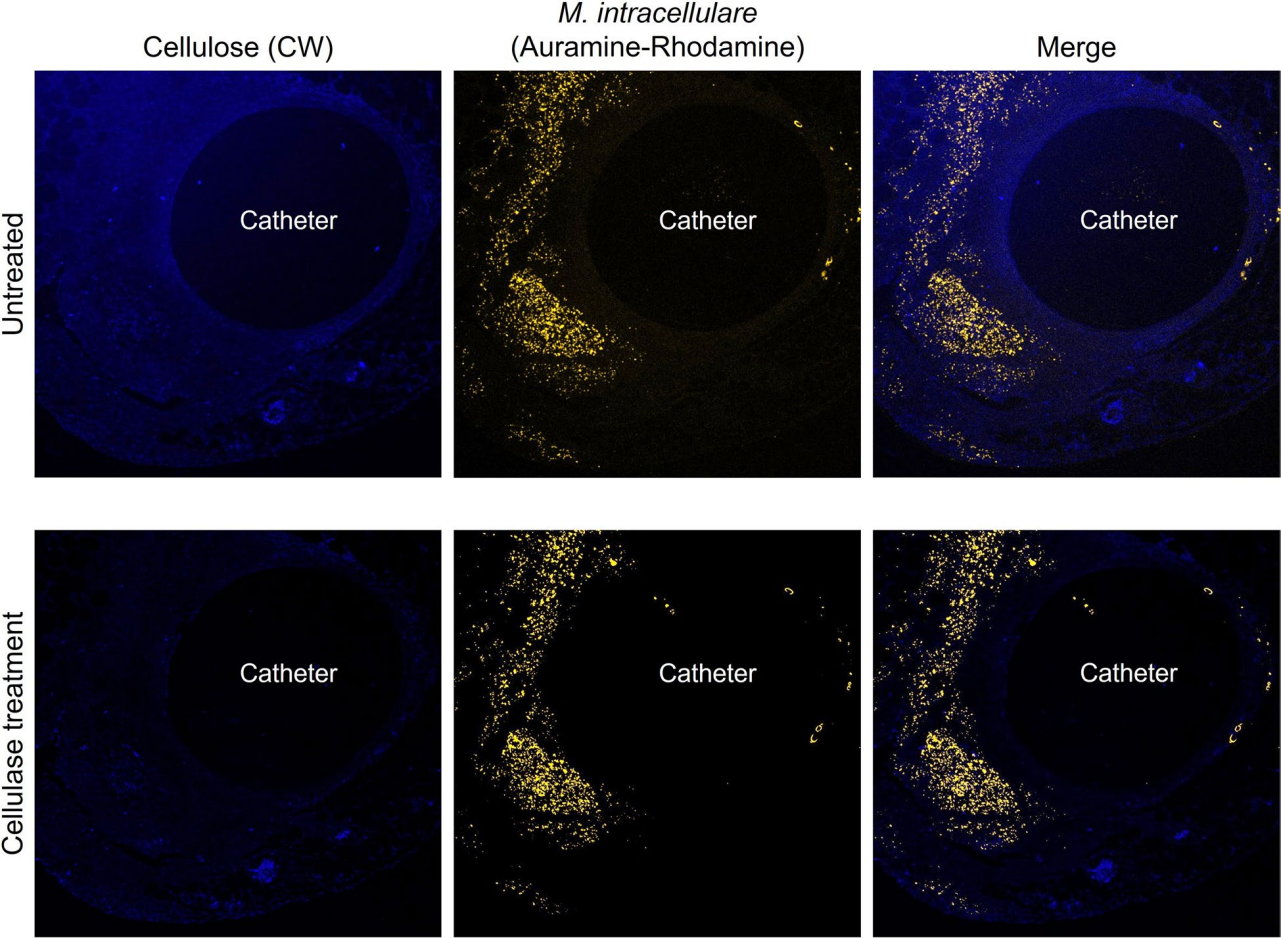
*M. intracellulare* formed cellulose-layer in the tissue. Catheter-induced biofilm-like structures in tissue with necrotizing granuloma were visualized by staining cellulose with calcofluor white (left), and *M. intracellulare* bacilli were stained with auramine–rhodamine (middle). These images were merged (right). The lower panel shows the stained images of cellulase-treated tissue.

Mycobacteria, including *Mtb* and *M. avium*, are known to form cellulose-based biofilms. We constructed *M. intracellulare* biofilms on plastic surfaces in vitro and stained them with calcofluor white, which specifically stains cellulose, to demonstrate the presence of thick biofilms (Supplementary Fig. S3). To determine whether *M. intracellulare* formed biofilms in the peritoneal cavity of untreated mice, the deparaffinized specimens of a necrotizing granuloma including catheters were stained with calcofluor white (CW) and observed by confocal laser scanning microscopy (CLSM). Cellulose was observed everywhere in the tissue surrounding the catheter. In particular, it was most abundant in the deepest layer in contact with the catheter and in the outermost layer facing the body tissue (Fig. 4, upper panel). Bacterial cells were visualized by staining with auramine O–rhodamine B dye. This staining revealed that bacteria were most abundant in the layer slightly distant from the catheter-contacting layer, where foamy macrophages accumulated. In addition, treating these specimens with cellulase and staining them with CW revealed the presence of small amounts of cellulose on the tissue. The cellulase treatment did not hinder the staining and visualization of *M. intracellulare* (Fig. 4, lower panel). These results suggest that bacilli grown at the lesion site may form cellulose-containing biofilms.

### Cellulose-layer inhibited antimicrobials from damaging *M*. *intracellulare*

**Figure 5:**
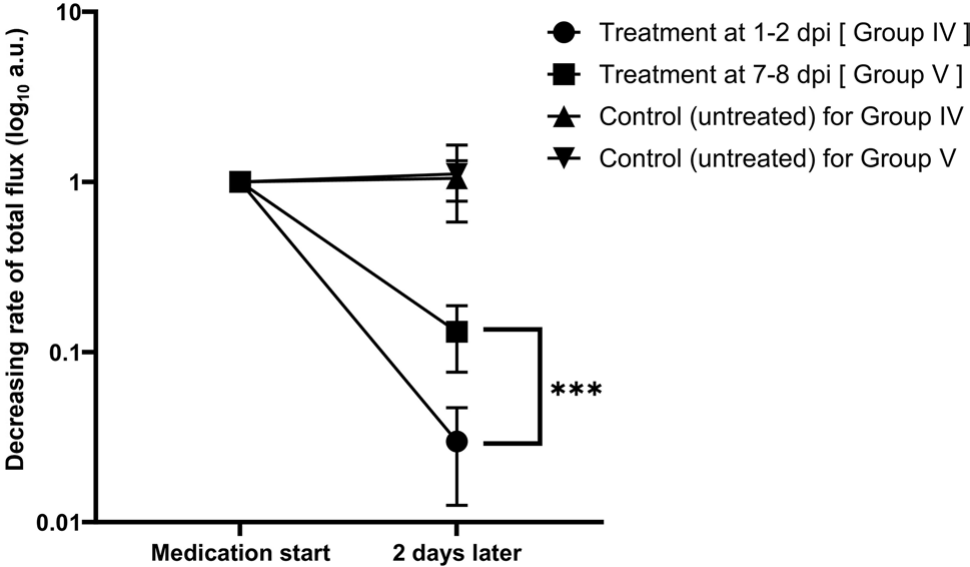
Tissue-associated biofilm-like structures attenuated the effects of medication therapy. The treatment effect was monitored using in vivo imaging by taking two different time points for the start of the medication (1 dpi and 7 dpi, see Fig. 1 Group III–V). In each group, the p/s value for pretreatment bioluminescent intensity was used as the baseline and the posttreatment intensity was plotted. Bioluminescence was detected at 3 dpi after drug-treatment at 1 − 2 dpi in Group IV (circles) and at 9 dpi after treatment at 7 − 8 dpi in Group V (squares). Triangles and inverted triangles represent the control (untreated) groups (Group III) for Group IV and Group V, respectively. Statistical analysis by ANOVA with Sidack’s multiple comparison test: ***P = 0.0007.

Given the previous identification of a cellulose layer, assumed to be a biofilm, we then examined whether biofilms of bacilli on the catheter suppressed the effectiveness of antimicrobials in inhibiting bacterial growth. The number of bacilli was expressed as bioluminescent intensity, and the ratio of change in total flux two days after treatment was plotted on a graph with the intensity of pretreatment as the reference. Mice were treated with triple drugs 2 days from 1 to 3 dpi (Group IV) and from 7 to 9 dpi (Group V; Fig. 1). Untreated mice served as controls (Group III) for both treatment durations. While the decreasing rate of intensity of the late treatment group significantly exceeded that of the early treatment group (13.2% in Group V *vs* 3.0% in Group IV; *P* = 0.0007; Fig. 5), the decreasing rate of intensity of the untreated group somewhat increased during both treatment periods but was insignificant.

## Discussion

Mycobacteria have innate multidrug resistance to antimicrobials and other harmful substances and can also easily acquire drug resistance. The formation of biofilm, a dense, intertwined matrix-like structure comprising proteins, polysaccharides, and extracellular DNA, can contribute to drug resistance in mycobacteria. The treatment of biofilm infections is often difficult because the bacteria in biofilms are highly resistant to antimicrobial agents and host defenses. The only effective treatment for patients with catheters, dialysis tubes, artificial joints, or cardiac pacemakers that are infected with bacterial biofilm is the removal of these implanted medical devices, which not only imposes a physical burden on the patient but also impacts the healthcare economy. Incidentally, approximately 60% of hospital-acquired infections are caused by bacterial biofilms on indwelling medical devices^37^. Although mycobacterial biofilm infections are not as common as the infections caused by *Staphylococcus aureus*, mycobacteria are extremely difficult to eliminate owing to their synergy with innate drug resistance. Therefore, we developed a mouse model of refractory biofilm infection of indwelling catheters caused by NTM bacilli.

We constructed bioluminescent *M. intracellulare* for the in vivo imaging of mice, which is derived from products of the *luxCDABE* genes of *Photorhabdus luminescens*. This bioluminescent system has several advantages. First, as with other bioluminescence systems, the tissue background level is extremely low, and the sensitivity is 10 times that of fluorescent proteins^38^. Second, because no excitation light is required, the system can be used at any experimental time without concern for phototoxicity or bleaching. Finally, because bioluminescence depends on the host’s metabolic energy, only live bacteria with active metabolism can be detected, reducing artifacts such as the observation of dead bacteria or debris. Although luciferase luminescence usually requires the administration of a substrate such as D-luciferin, luciferins are metabolized very rapidly in vivo, and most luciferase signals exceed their peak within a few minutes^39–41^. Therefore, time management during experiments is extremely complex and often leads to large variations in data. This study’s Lux bioluminescence system can resolve the above problems because the lux operon provides what is needed for luminescence. With its high sensitivity and dynamic range, the IVIS in vivo imaging system can detect and track the dynamic process of NTM infection and compare photons emitted per unit time with very low error^42^. Bioluminescence imaging revealed that *M. intracellulare* grew dispersedly around the indwelling catheters in mice. The dispersed bacteria in the early infection gradually decreased with time, and the bacteria survived in another area of the peritoneal cavity. These data suggest that the plants of bacterial production were established around the catheter and that the proliferating bacteria were pumped to each area. The antimicrobial treatment eliminated bacteria that had spread into the abdominal cavity, whereas catheter-attached bacteria survived longer, suggesting that either these bacteria form biofilm-like structures that act as barriers to antimicrobial permeation or they develop a more drug-tolerant phenotype as they adapt to survive in this extracellular matrix (ECM). This mouse model mimicked catheter-associated systemic disseminated NTM disease by allowing the bacteria to remain in mice for a longer time than the time these bacteria remain in the general abdominal infection model. This model may also be adapted as the simplified in vivo antimicrobial evaluation system against NTM biofilm infection within a few weeks.

The C3HeB/FeJ mouse is often used in tuberculosis studies that reflect human-like pathology, but its usage in infectious disease studies of other mycobacteria is uncommon^34,43^. An important characteristic of this mouse is that mycobacterial infection causes pathological states such as caseous necrosis and abscesses. We infected the peritoneal cavity of C3HeB/FeJ mice with *M. intracellulare* and performed necropsy. Dome-like or sac-like raised structures were found throughout the tissues. Although the number of neutrophils and areas of necrosis were slightly lower than in fulminant catheter infections caused by other bacteria such as *Staphylococcus aureus*, a large amount of yellowish-white pus was detected, suggesting that these structures were *M. intracellulare* infection-induced abscesses^44,45^. The formation of severe abscesses was observed, particularly in the tissue surrounding the indwelling catheter. Bacteria colonizing these sites were less susceptible to drugs and persisted in the body for longer periods, suggesting that these sites served as protective barriers for these bacteria. Because abscesses and live bacteria were also detected in the spleen and mediastinal lymph nodes of the proposed model, the bacteria may have spread from these nests throughout the body. Pathological analysis revealed fibrotic cells in the tissue in contact with the catheter, with, or without treatment. This was consistent with a nonspecific inflammatory profile resulting from the catheter's surgical implantation^44,46,47^. A few neutrophils and areas of necrosis were also observed in the treated group. The in vivo imaging analysis revealed that bacteria around the catheter remained alive in the mouse peritoneal cavity for a prolonged period, even after medication treatment. As a result, the above-described host immune response to infection was most likely present in the treated group's proximal catheter tissues. However, the number of visible bacteria in the untreated group was very high, with cellular fibrosis, and an increase in macrophages, whose response to bacterial infection was remarkable. These data strongly represent the characteristics of medical device-associated infections caused by bacteria, particularly mycobacteria, and demonstrate that complete bacterial neutralization is extremely difficult.

Mycobacteria secrete abundant cellulose during biofilm formation, which is assembled into a biofilm as part of an intricate barrier structure. Currently, cellulose is the only mycobacterial biofilm-specific marker, given that planktonic bacteria do not produce it^17,48^. We examined whether *M. intracellulare* formed biofilms in mice by staining surrounding tissues, including catheters, with CW, and auramine O–rhodamine B dyes. These results suggested that the mycobacteria-formed biofilm-like structures including cellulose was widespread surrounding the catheter. Cellulose was also degraded by cellulase treatment, indicating that the ECM of NTM is highly sensitive to cellulase treatment. Therefore, exposing the biofilms to cellulase may help in disrupting the structure of biofilm by degrading cellulose, a component of the biofilm. Biofilm formation on the surfaces of indwelling medical devices such as catheters is thought to trigger infections associated with these devices. Several reports have shown NTM biofilm formation in mammals, but these were only indirect predictions based on structural studies from electron microscopic observations^49,50^. This study showed that *M. intracellulare* could form biofilms in vivo not only on the surfaces of artifacts but also in the surrounding tissues. However, as mycobacterial biofilms are currently detected solely through cellulose staining, further research, including the development of different biofilm-specific markers, is needed. As with other bacteria such as *Staphylococcus aureus*, *Mtb* can also acquire drug resistance by forming biofilms^51,52^. NTM-formed biofilms are expected to contribute to drug resistance in the same way as *Mtb*, although this has never been proven. We also suggested that biofilms reduced medication efficacy by testing the rate of decrease in NTM bacilli counts when treatment initiation was delayed. Indeed, antimicrobial susceptibility can be influenced not only by biofilm formation but also by acquired antimicrobial resistance. Typically, bacteria acquire resistance to antimicrobials either after extended exposure to these drugs at low concentrations in vitro or following prolonged antimicrobial treatment in vivo^53,54^. In this study, antimicrobials were administered only twice within a short period. It is considered highly unlikely for individual bacteria to acquire drug resistance during this short period. Therefore, this study represents the first instance of indicating that NTM bacilli could form biofilms in vivo and that this process reduces the therapeutic efficacy.

In conclusion, we established a mouse model of systemic disseminated *M. intracellulare* infection that mimicked catheter-related infections. We also suggested that *M. intracellulare* may form a biofilm on the catheter and disseminated to surrounding tissues, thereby weakening the therapeutic effect of medication. This proposed model may allow us to evaluate the efficacy of anti-NTM or anti-NTM biofilm drugs. Further in vitro and in vivo studies are required to uncover what triggers NTM to form biofilms, whether they are associated with virulence, and how they evade host immunity. It is also crucial to investigate whether the pathogenesis with biofilm and abscess formation in this study can be observed in other NTMs, and this study can yield interesting results. Overall, our data suggest that NTM may form biofilms at all infection sites, such as the respiratory organs and skin. Therefore, the removal of biofilm on prosthetic materials and tissues with enzymes or antimicrobial peptides may be a promising target for novel treatments of NTM disease.

## Materials and methods

### Strains and culture conditions

*Mycobacterium intracellulare* subsp. *intracellulare* (ATCC13950) type strain was provided as JCM6384 by Japan Collection of Microorganisms, RIKEN BRC, which is participating in the National BioResource Project of the MEXT, Japan. This strain was grown at 37 °C under shaking conditions in enriched 7H9 medium (Middlebrook 7H9 medium supplemented with 0.2% glycerol, 0.05% Tween-80, and OADC-enrichment) or on enriched 7H10 agar (Middlebrook 7H10 supplemented with 0.5% glycerol and OADC-enrichment). Whenever necessary, 25 µg mL^−1^ kanamycin was added to the medium for clone selection.

### Animals

Mice were housed within the animal biosafety level 2 containment facility at Leprosy Research Center of National Institute of Infectious Diseases at 21 °C ± 2 °C room temperature, 40–60% humidity, on a 12 h light–dark cycle (8 a.m. to 8 p.m.), and given food and water ad libitum, according to institutional guidelines. All animal experimental protocols were reviewed and approved by the National Institute of Infectious Diseases' Animal Use and care committee (Permit Number: 121014, 121134), and all methods were conducted in accordance with the relevant guidelines and regulations, and we followed the recommendations outlined in the ARRIVE guidelines for conducting research on animals.

### Antimicrobials

The following reagents were used: clarithromycin (FUJIFILM Wako Chemicals Co., Osaka, Japan); ethambutol (MilliporeSigma, MA., USA); and rifampicin (DAIICHI SANKYO HEALTHCARE CO., LTD., Tokyo, Japan). These stock solutions were prepared by dissolving them in sterilized water. Aliquots were distributed and stored at − 30 °C.

### Construction of plasmids encoding LuxCDABE

The *luxCDABE*-coding region was amplified by PCR, with the sense primer 5′-CTGAGGAGGAATCTCCATATGGCAAATATGACTAAAAAAATTTCATTC-3′ and the antisense primer 5′- GTCGATCGTACGCTAGTTAACTCAACTATCAAACGCTTCGGTTAAGC-3′, using pLUX (gift from Dr. Kaneyoshi Yamamoto at Hosei University)^55^ as a template to introduce *Nde*I and *Hpa*I sites at their 5ʹ and 3ʹ ends, respectively. The resulting fragment was cloned between the *Nde*I and *Hpa*I sites of the pKRB32 vector^56^, which is a pMV361-type *E. coli*-*Mycobacterium* shuttle vector that carries the *hsp60* promoter, integrase gene, and kanamycin resistance gene^57^, to yield plasmids encoding LuxCDABE (pKRB129).

### Mouse model

Specific pathogen-free C3HeB/FeJ mice (6–10 weeks old, female) used in this experiment were purchased from Jackson Laboratories (Bar Harbor, ME, USA) and bred. The 24-gauge catheter (TOP Corporation, Tokyo, Japan) was cut to 10 mm. These two fragments were implanted into the right abdominal region of each mouse under anesthesia (100 μL/mouse, medetomidine (NIPPON ZENYAKU KOGYO Co., Ltd., Fukushima, Japan), 20 μg/mL; midazolam (Maruishi Pharmaceutical Co., Ltd., Osaka, Japan), 600 μg/mL; butorphanol (Meiji Animal Health Co., Ltd., Kumamoto, Japan), 1 mg/mL), and the wound was closed with clips for animal surgery (CellPoint Scientific, Inc., MD, USA). After a week, the mice were infected by i.p. injection of *M. intracellulare* (10^8^ CFU/mouse), which was grown in the tween-free 7H9 medium under shaking conditions at 37 °C. Following infection, the mice were randomized into the following five subgroups (Fig. 1).

Group I: No antimicrobial treatment (control group) for 22 days.

Group II: Treatment with triple-drug therapy of clarithromycin + ethambutol + rifampicin 5 days per week for 22 days.

Images of the mice were acquired weekly until 22 dpi using IVIS in vivo imaging systems.

Group III: No antimicrobial treatment (control group) at 1 − 9 dpi.

Group IV: Treatment with triple-drug therapy at 1 − 2 dpi.

Group V: Treatment with triple-drug therapy at 7 − 8 dpi.

Antimicrobials were administered by oral gavage in all groups. The drug doses were 10 mg/kg/day for rifampicin and 100 mg/kg/day for clarithromycin and ethambutol. Images of the mice were acquired at 3 dpi (Group IV) or 9 dpi (Group V).

These studies were performed in triplicate.

### In vivo imaging and analysis

Anesthetized mice were laid in a closed chamber, which maintained clean air with a membrane filter (0.22 μm pore size) designed by Yamazaki Seisakusho Ltd. (Tokyo, Japan), and imaged via IVIS Lumina LT (PerkinElmer Inc., MA, USA). The images were acquired in an exposure time of 300 s and filtered using the smoothing algorithm (5 × 5 pixels) to remove signal noises and set uniform color scales. We defined a contour region of interest, with the threshold appropriately adjusted. In addition, mice were imaged via CosmoScan FX (Rigaku, Tokyo, Japan) using the following parameters, 90 kV, 88 μA, and 45 mm FOV, resulting in a scan time of 2 min.

### Histological analysis and staining

All tissue and abscess samples were fixed with a 10% neutral buffered formalin solution. Samples were paraffin embedded, sectioned to 5 μm thick each, and stained with hematoxylin–eosin, and Fite-Faraco by Advantec Co., Ltd. (Osaka, Japan). The paraffin sections were deparaffinized with PathoClean (FUJIFILM Wako Chemicals Co., Osaka, Japan) and washed in serial dilutions of ethanol to rehydrate the tissue. The deparaffinized samples were stained with the auramine–rhodamine staining kit (MilliporeSigma, MA., USA) for acid-fast bacilli staining. For cellulose-specific detection, the samples were stained with 0.1% calcofluor white solution (MilliporeSigma, MA., USA) for 15 min and placed in running tap water for 5 min. Calcofluor white binds to β liked polysaccharide, such as cellulose and chitin, and cannot penetrate intact cell membranes. The stained samples were observed using confocal laser scanning microscopy (Carl Zeiss LSM900, Oberkochen, Germany) with a 10 × /0.45 NA objective lens, a 405-nm 5-mW diode laser (Excitation Wavelength: 254 nm, Emission Wavelength: 432 nm) for calcofluor white and a 561-nm 10-mW diode laser (Excitation Wavelength: 543 nm, Emission Wavelength: 565 nm) for auramine–rhodamine, and the Airyscan2 Multiplex was used to obtain superresolution images.

### Cellulase treatment

The serial sections of fixed tissue samples were deparaffinized. The samples were then treated with 5 mg/ml cellulase derived from *Trichoderma viride* (MilliporeSigma, MA., USA) in 0.05 M phosphate-citrate buffer (MilliporeSigma, MA., USA) for 6 h at 37 °C under humid conditions. The treated samples were washed under running water for 10 min and then stained with the auramine–rhodamine and calcofluor white, as described in the previous section.

### Statistical analysis

The resultant flux values of bioluminescent intensity were reported as the mean ± standard deviation (S.D.). The significance of each experiment was determined by analysis of variance (ANOVA) with Sidack’s multiple comparison test using GraphPad Prism 9 (GraphPad Software, San Diego, CA).

### Supplementary Information

Supplementary Information.

## Data Availability

All data generated or analyzed during this study are included in this published article.
