# Supplementary material for: Catheter-associated Mycobacterium intracellulare biofilm infection in C3HeB/FeJ mice
Source: Sci Rep. 2023 Oct 10;13:17148. doi: 10.1038/s41598-023-44403-0 (PMC10564925; doi:10.1038/s41598-023-44403-0)
Supplement: Supplementary file 1 — Supplementary Information. [file 41598_2023_44403_MOESM1_ESM.pdf]

Supplementary Information

Catheter-associated *Mycobacterium intracellulare* biofilm infection in C3HeB/FeJ mice

Kentaro Yamamoto[1*¶], Yusuke Tsujimura[1¶], Manabu Ato[1]

[1] Department of Mycobacteriology, Leprosy Research Center, National Institute of Infectious Diseases, Aoba-cho, Higashimurayama, Tokyo, Japan

[¶]Authors contributed equally to this work.

*Corresponding author

E-mail: keyam@niid.go.jp

Fig. S1

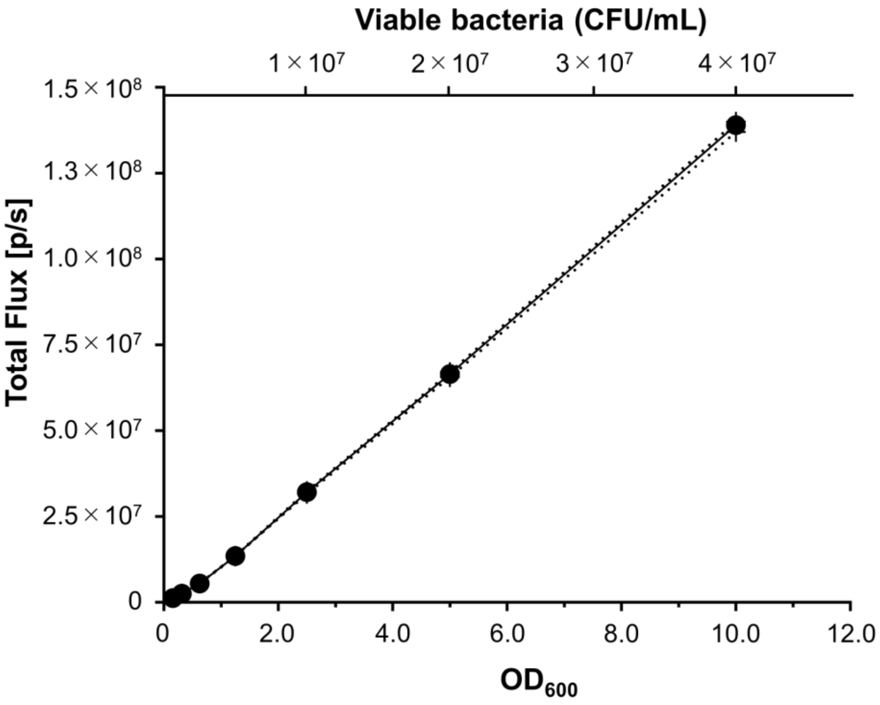

Figure S1 | Correlation between the total flux and the number of bacteria

Precultured bacterial cells in the midlogarithmic phase were concentrated at OD_{600}=10.0 using an enriched 7H9 medium. The cells were diluted in twofold series and inoculated in clear flat-bottom 96-well black plates (Corning Inc., NY, USA) at a total volume of 200 µL per well. Bacterial bioluminescence was detected using IVIS Lumina LT (PerkinElmer Inc., MA, USA). Diluted cells were cultivated on selective Middlebrook 7H10 agar plates supplemented with 10% OADC, which were incubated at 37°C for 2 weeks before determining CFU counts.

Bioluminescent intensity as photons per second (p/s) and the number of bacterial cells were calculated and plotted. The linear regression ($r^2 = 0.9994$, $p < 0.0001$) represented by the solid line (closed circle) and each experiment data are represented by the dotted lines (cross symbol) correspond to each experiment.

Fig. S2

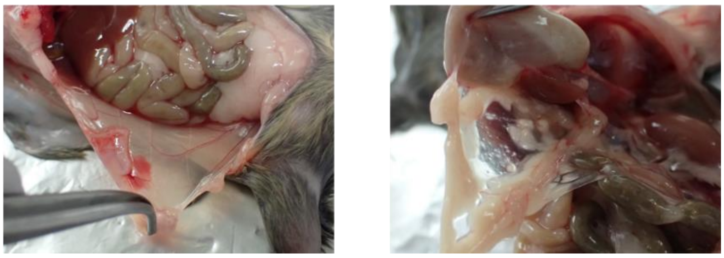

Figure S2 | Postinfection abdominal necropsy in mice without indwelling catheters

Mice were infected by i.p. injection of *M. intracellulare* (10^8 CFU/mouse), which was grown in the tween-free 7H9 medium under shaking conditions at 37°C and adjusted with PBS. Mice were euthanized and abdominal necropsy was performed three weeks after infection. Few infection-related conditions, such as abscesses, were observed.

Fig. S3

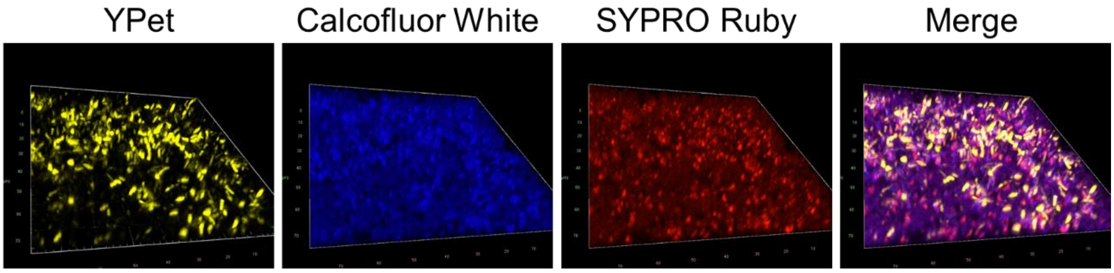

Figure S3 | Biofilm formation of *M. intracellulare in vitro*

The YPet-coding region was amplified by PCR using pYPet-His (Addgene #14031). The resulting fragment was cloned into the pKRB1 plasmid (Yamamoto *et al.*, 2021) to yield plasmids encoding YPet (pKRB88). The strain carrying pKRB88 was grown at 37°C under shaking conditions in an enriched 7H9 medium with 25 µg mL^{-1} kanamycin. Precultured cells in the mid-logarithmic phase were diluted at OD_{600} = 0.2 with an enriched 7H9 medium. Adjusted cells were inoculated into clear flat-bottom 96-well plates (CellCarrier-96 Ultra, Perkin Elmer) with 6 mM DTT in a total volume of 200 µL per well. Assay plates were incubated for 2 weeks at 37°C under stationary conditions. Attached biofilms were washed three times with PBS containing 0.05% Tween-80 (PBS-T), and stained with calcofluor white (for cellulose) and SYPRO Ruby (for proteins). Biofilms were analyzed using confocal microscopy with Airyscan2 Multiplex to obtain superresolution images.

Supplementary Table

	MIC (μg mL^{-1})		
	CAM	EMB	RIF
WT	0.03	2	16
pKRB129	0.03	2	8

Supplementary Table | Antibiotic susceptibility analysis

The integrated vector encoding LuxCDABE was expressed in the *M. intracellulare* subsp. *intracellulare* (ATCC13950) type strain. This analysis was performed in triplicate. Abbreviations: CAM, clarithromycin; EMB, ethambutol; RIF, rifampicin.

The minimum inhibitory concentrations (MICs) were determined using a twofold serial dilution method. Precultured cells in the mid-logarithmic phase were diluted to achieve the OD_{600} of 0.1 using an enriched 7H9 medium. In a clear flat-bottom 96-well plates, 100 μL of diluted cells were inoculated in each well containing 100 μL of cation-adjusted Mueller–Hinton medium supplemented with 5% OADC with the assay compounds. Assay plates were incubated for 3 weeks at 37°C under stationary conditions. The MIC for each strain was defined as the lowest concentration of a drug needed to inhibit bacterial growth via visual observation.
